# Case of Extensive Suppuration, Attended with Uncommon Appearances, Terminating Successfully

**Published:** 1804-09-01

**Authors:** John Moodie


### CASE OF EXTENSIVE SUPPURATION, *attended with uncommon Appearances, terminating successfully; communicated by* JOHN MOODIE, M. D.

JOHN SKRINE, a man bred to farming business, naturally of a robust and healthy constitution: but from the information of his apothecary, it appeared that he had lately suffered much from syphilitic infection. The 18th of September, 1801, he consulted me respecting a painful affection, which he had for some days felt about the top of of his right shoulder, which, he said, his friends persuaded him was rheumatic gout, and advised the use of the hot-bath, or dry pumping applied to the part affected.

Upon examination, I found an enlargement possessing the whole articulation of the *humerus*, attended with redness, throbbing, tumour, increased action of the vessels, tension, and impaired strength of the arm. His cheeks were alternately flushed, with frequent severe rigors, and other symptoms indicating a tendency to suppuration. His tongue white, skin hot and dry, with great thirst, flatulence, and acidity at stomach. Pulse hard and full, belly costive, his urine high coloured, and voided in small quantities.

On consideration of the above symptoms, I was apprehensive that the suppurative process had already begun, or was unavoidable, and that it would be impossible to effect a cure by resolution.

Having cautioned him against the use of the pump, a gentle laxative was directed to be taken occasionally; and a saline mixture with the *pulvis antimonialis*, of the London Dispensatory, two large spoonfuls every three or four hours, with an opiate at night.

The patient living at some distance from my residence, I heard nothing further from him till the 23d, when he again personally applied to me; I desired to know if he had followed the directions previously given? He seemed desirous to conceal what had been done, but acknowledged, that, at the urgent solicitation of his friends, he had been induced to try the *metallic tractors*, but finding no relief from that expedient, he next had recourse to the hot-bath, where he received 150 strokes of the pump,* chiefly directed to the shoulder and side affected.

Upon examination of the parts, and the patient, respecting the effects produced upon the system, during this last dangerous experiment; it appeared evident, that the force of the falling column of water on the inflamed surface, while he was labouring under a symptomatic fever, occasioned faintness and other unfavourable symptoms, which had nearly proved fatal to him during the operation.

It

* The degree or quantity of the application, improperly denominated dry pumping, is measured by the number of times the handle of the pump is raised and thrust down while the patient is exposed to the stream of the water.

It has already been observed, that the appearance of the tumour, when first examined, was comparatively small and circumscribed, occupying chiefly the articulation of the *humerus.* But the mechanical violence of the pump, by adding to the stimulus, and relaxing the solids, occasioned an extensive process of the secretion of the fluids into the surrounding cellular membrane, which greatly increased its size; insomuch, that the whole surface, from the top of the shoulder to the spine of the *ileum,* was in a state of inflammation, irritation, and extreme pain, from which he had not enjoyed a single interval of ease during the night. His skin was hot and dry, pulse 128, with head ach, sickness at stomach, and diarrhœa.

The pain being thus extreme, with increase of tumour, sense of weight and throbbing, the parts more softened and pointed; it now became necessary to hasten the effused fluid into pus, as well as to relax the surrounding integuments, with a view to promote the most favourable directions of the abscess. For this purpose, fomentations were ordered to be used twice a day, to alleviate the pain, and emollient cataplasms applied warm and sufficiently large to cover the parts. And as danger was evident, he was desired to comply strictly with the injunctions which were given, to keep himself quiet, and at home.

The saline mixture with the *pulvis antimonialis* was continued, till the febrile symptoms were removed; and to check the diarrhœa, the following medicine was ordered.

℞. Pulveris cretæ compositi cum opio ʒij. Tincturæ cinnamomi compositæ ℥j. Aquæ menthæ sativæ ℥v. M. sumat cochl. ij. ampl. post singulas sedes liquidas.

To procure rest, and a copious diaphoresis, the following was prescribed.

℞. Tincturæ opii guttas lxx. Spiritus ætheris vitriol. comp. Vin. antimonii tartarisati aa. ʒj. Aquæ pimento dilutæ ℥iſs. f. haust. quotidie nocte sumendus.

The 25th during the night he perspired freely, and next morning the febrile symptoms were considerably abated. The saline mixture above mentioned was repeated, which kept up a gentle perspiration, and the diarrhœa was soon removed by the cretaceous mixture.

During the remainder of this month, he suffered much pain in the right shoulder and side; and notwithstanding the most cautious method of treatment, to which he submitted with great resolution and perseverance, the symptomatic fever frequently returned, attended with profuse sweats, and he became so much emaciated, that every attempt tempt to move himself in bed occasioned much pain, perpetual anxiety, and restlessness. And although opium was exhibited in uncommonly large doses, yet want of sleep rendered his situation truly deplorable.

His appetite, nevertheless, was good; wine, broths, and jellies, were added to his diet, and the maturation of the abscess was promoted by every possible means. But still it did not point to any particular part.

In this situation he remained till the 2d of November, when there appeared an evident fluctuation of *pus*. It was therefore my opinion, that the tumour should be immediately opened, and a surgeon requested to attend for that purpose. But, dreading the operation, the patient refused to see any other professional gentleman. Having met the apothecary next morning, who observing the magnitude of the abscess, he declined opening it, being apprehensive that the patient might sink under the immensity of the discharge. I therefore determined to perform the operation myself, which was immediately done with a scalpel, at the lower and most depending part of the tumour, where it pointed about four inches below the right breast. The quantity instantly discharged was upwards of four quarts, of yellowish, opaque, thickish, and almost inodorous fluid; and it may be supposed, that, at least a pint and a half issued from the orifice for several days following.

After the abscess was opened, the violence of his pain began sensibly to diminish, his pulse became softer and more regular, and partial remissions of fever ensued; I then ordered the *decoctum corticis Peruviani*, with the *conf. aromat.* and the *acid. vitriol. dilut.* Of this mixture a tea-cup full was taken every three or four hours, as his stomach would bear it. And good effects were obtained in procuring rest, by means of opium combined with some grateful aromatic.

About the 6th, the patient seemed to regain strength, so that he was enabled to walk from his bed to the fire, and sit up for several hours in the day. His appetite was equal to what it had been when in health. And he thus continued mending about a fortnight, when he suddenly complained of pain in his loins and hips, (owing to cold,) which again confined him to his bed. The first week after the tumour was opened, the discharge was profuse, thin, and putrid, excoriating the edges of the aperture, destroying integuments, and bringing along with it considerable portions portions of sloughy cellular membrane, which sometimes choked up the opening, and obstructed the drain.

The integuments of the right side were œdematous, detached from the ribs and spine, and in some parts had the appearance of an empty bag; in others there was an evident fluctuation of pus, apparently in a state of maturation. And as there appeared to be a channel, or communication, between the different cysts, I had therefore reason to suppose, that the whole would be collected, and form one abscess of considerable extent.

The 15th he was suddenly seized with a pain in his left side, cough, and difficult respiration. I prescribed nitre with camphorated tincture of opium, to promote expectoration; a blister was applied to his side, and the following mixture, which I have often found of eminent service in relieving those distressing symptoms.

℞. Ammoniæ præparatæ ℈iß. Aquæ puleg. ℥ij. Aquæ pimento dilutæ aa. ℥iij. Sacchari purificati ℈iij. Olei olivæ ℥j. M. Sumat cochl. ij. ampl. quarta quaque hora.

By using the above medicines for two or three days, his cough was much better, and he continued the bark mixture as before. And to procure rest, the opiate was gradually increased to upwards of half an ounce of *tinct. opii cum mist. camphor.* ℥jß. *Spt. æther. vitriol. comp. guttas lxxx. singulis noctibus.*

Another large abscess having formed on the right side, about four inches above the *spine* of the *ilium*, it was opened on the 19th, whence issued nearly five pints of well digested pus. On the morning of the 21st, a tumour, of considerable size, was observed on the lower part of the *sternum*, near the *cartilago ensiformis*; this was opened on the 25th, and discharged about two quarts.

The right shoulder was much inflamed and painful, and two days after, an abscess pointed about five inches below the right breast, which burst in the night, and about two quarts of *pus* were supposed to have issued from the orifice. This continued open near a week, during which the acrid humour destroyed a considerable portion of the surrounding integuments.

The 29th, an abscess of uncommon magnitude appeared between the shoulders, resting upon the superior *vertebræ* of the back. This was so extremely painful that the patient could not for forty-eight hours find ease in any situation; he was therefore supported by pillows, in a supine posture. This tumour was opened the 6th of December, and and evacuated upwards of four quarts of fluid, of a purulent consistence, tinged with blood, and the orifice was closed three days after. When another abscess (being the fifth) came to suppuration upon the *lumbar vertebræ*, between this last and most of the former, there was a perceptible communication. The 17th it was opened, and discharged about four pints and a half of pus tinged with blood.

About this time his cough returned, with oppressed respiration, pain in his side, and diarrhœa; skin hot and dry. In order to promote expectoration, the following decoction was directed; and, to relieve the purging, he occasionally took two spoons full of the cretaceus mixture.

℞. Radicis senekæ contusæ, ℥ß. Aquæ fontanæ, ℔iß. Decoque ad ℔i. et cola.

℞. Supra præscript. ℥v. Aquæ ammoniæ acetatæ ℥ij. Spiritus pulegii, ℥i. M.

During the night he had several hours refreshing sleep; his cough easier, expectoration less difficult, the pain in his side abated, his pulse soft and less frequent, with a gentle perspiration, and his urine deposited a considerable sediment.

The patient having taken the bark for some time, as often, and in as large quantities, as it could be given with safety, he now became averse to that medicine in any form whatever. I therefore ordered the *decoctum sarsaparillæ cum pulv. ejusdem, dosis, libra dimidia, ter quotidie*, and recommended a milk diet to correct the acrimony of the purulent discharge.

The symptomatic fever, however, continued with fewer remissions. The lower extremities became anasarcous, with a tendency to colliquative sweats, and considerable emaciation. And, although the discharge from the last abscess had totally ceased, it was nevertheless obvious that the patient lost strength daily. His pulse was frequent and tremulous, varying from 120 to 130, upon the slightest exertion or agitation of body.

Under these circumstances, I urged him to resume the bark. Collections of pus doubtless implies a certain degree of inflammation, but the accompanying symptoms prove it to be of that irritable kind arising from an unequal determination of the blood attendant on great debility. In such cases, therefore, the use of the bark is admissible, at the same time that our best endeavours should be exerted to support and strengthen the system by means of other powerful tonics.

Some have affirmed, that the Peruvian bark has been known to occasion difficult respiration in such cases, and on that account considered in the light of an uncertain and hazardous remedy. Authorities, however, are not wanting in favour of this remedy, when prudently administered. Besides, Morton, who extols it highly, we have the testimony of Sir John Pringle in its favour. He remarks, that he had frequently given three or four spoonsfull of decoction of bark twice a day, without observing that it heated the system, or obstructed the breathing; but, on the contrary, that it had good effects when the patient complained of low spirits and weakness.*

If the quantity of the remedy used by this author be thought too inconsiderable to afford any conclusion in its favour, it may be proper to refer to an account of several very alarming cases of pulmonary affections successfully treated by a more liberal exhibition of bark some time ago, published in the Medical Communications.†

Catarrh, a disease much connected with consumption, may be also adduced in support of the utility of the bark in cases of suppuration. Chronic catarrhs are sometimes extremely troublesome, by which the patient is considerably reduced, and it becomes necessary to employ the bark with a view to strengthen the constitution and diminish the discharge, by increasing the tone of the vessels distributed to the mucus membrane of the trachea. This may be further illustrated by a late author of reputation, who has written on the Materia Medica.‡

As the patient could not be prevailed upon to continue the bark, and as he was daily losing strength, his cough at times troublesome, respiration somewhat difficult, the lower extremities bloated, and a slight degree of anasarca extended to the rim of the belly; on the 27th, Dr. A. Fothergill,

---

* Army Diseases, Third Edition, p. 164, Note.

† See Medical Communications, Vol. 1, p. 260.

‡ "Binos ipse ab empyemate chinchina (i. e. cort. Peruv.) curavi. Quo magis sputa fætent, eo certior cura. In phthisi pulmonali sapè quidem præclara præstat, sæpé autem nihil efficit. Quando apthæ accesserunt, symptomaticæ in hoc morbo, evidenter nocuit, nisi sputa suppressisse diceres. Certè, ubi sputa in phthisi nimis abundant, cortex indicatur; si verò, cum oppressione pectoris, subitò diminuantur, cortici non inhærendum. Nullam vidi noxam ex moderata dosi chinchinæ quotidiè sumpta in phthisi, etiamsi sanguis, per venam sectam emissus, crustâ inflammatoria obductus subinde fuit." Vide Bergius Materia Medica, tom. 1. p. 109.

Fothergill, an eminent Physician in this city, visited him at my request, and the following medicines were directed.

℞. Myrrh. opt. ʒi. G. oliban. ℈ij. Syr. balsam. q. s. ut f. pil. xx. capiat. iij. Nocte maneque cum haust. infus. sasafras tepid.

℞. Tinct. opii camph. ℥i. capiat cochl. ij. minim ex haust. infus. sasafras. post singul. dos. pilul.

Having taken the above medicines for a short time, they seemed to have a good effect, but were afterwards changed for the bark in substance, of which he took a drachm three or four times a day in port wine, and persevered in the use of it about three weeks. When, notwithstanding his habitual excess in drinking, by the middle of January, 1802, he was perfectly restored to health, except a slight degree of lameness about the articulation of the shoulder.

Although this patient had no apparent symptoms of the disease when he complained of the pain about his shoulder, yet there cannot be a doubt that it arose from venereal infection. For, it is well known, that inflammation from the above cause has a tendency to suppurate, and generally does so, unless the virulence of the disorder is destroyed by mercury, or a revulsion is produced by some other disease, or by evacuant medicines.

The quantity of purulent fluid, clearly ascertained to have been discharged, on opening the abscesses, which successively formed (as above described) was upwards of *eighteen quarts*, exclusive of that which was removed with the dressings and by other means.

As supplying many of the inhabitants of this city with milk, this man is well known; and he lately informed me, that since his recovery as abovementioned, he has enjoyed an uninterrupted state of health, and is now entirely free from lameness.

*Bath, August* 14, 1804.

